# Supplementary material for: Heart rate variability and psychosocial symptoms in adolescents and young adults with cancer
Source: PLoS One. 2021 Nov 4;16(11):e0259385. doi: 10.1371/journal.pone.0259385 (PMC8568181; doi:10.1371/journal.pone.0259385)
Supplement: S1 Table — Association of patient reported anxiety and depression with HRV measures stratified by gender, cancer type, and age using linear regression models. Models adjusted for age and cancer type when not the stratum of interest. Coefficients interpreted as change in PRO score for every 10msec change in HRV. CNS and non-CNS solid tumor categories collapsed for analysis. (DOCX) [file pone.0259385.s001.docx]

| Supplemental Table 1. Association of Heart Rate Variability with Depression and Anxiety Stratified by Sex, Cancer Type and Age | | | | | | |
| --- | --- | --- | --- | --- | --- | --- |
|  | **SDNN** | | | **RMSSD** | | |
|  | β coefficient | 95% CI | p-value | β coefficient | 95% CI | p-value |
| *Anxiety* |  |  |  |  |  |  |
| *Males* | 0.11 | [-0.30 - 0.52] | 0.59 | 0.02 | [-0.39 - 0.43] | 0.93 |
| *Females* | 0.29 | [-0.37 - 0.95] | 0.37 | 0.48 | [-0.25 - 1.21] | 0.19 |
| *Leukemia/Lymphoma* | 0.08 | [-0.33 - 0.49] | 0.69 | 0.06 | [-0.38 - 0.50] | 0.79 |
| *Solid Tumor* | 0.51 | [-0.37 - 1.38] | 0.24 | 0.51 | [-0.29 - 1.31] | 0.19 |
| *12-15y* | -0.03 | [-0.55 - 0.49] | 0.91 | 0.17 | [-0.41 - 0.74] | 0.56 |
| *16-19y* | 0.35 | [-0.25 - 0.95] | 0.24 | 0.22 | [-0.39 - 0.84] | 0.47 |
| *20-25y* | 0.82 | [-0.73 - 2.38] | 0.24 | 0.24 | [-1.32 - 1.81] | 0.72 |
| *Depression* |  |  |  |  |  |  |
| *Males* | -0.02 | [-0.47 - 0.43] | 0.92 | 0.03 | [-0.42 - 0.47] | 0.9 |
| *Females* | 0.37 | [-0.20 - 0.93] | 0.19 | 0.54 | [-0.08 - 1.15] | 0.09 |
| *Leukemia/Lymphoma* | 0.18 | [-0.18 - 0.55] | 0.32 | 0.25 | [-0.14 - 0.65] | 0.2 |
| *Solid Tumor* | 0.02 | [-0.90 - 0.93] | 0.97 | -0.006 | [-0.85 - 0.84] | 0.99 |
| *12-15y* | 0.05 | [-0.40 - 0.49] | 0.84 | 0.29 | [-0.19 - 0.78] | 0.23 |
| *16-19y* | 0.17 | [-0.43 - 0.78] | 0.56 | 0.06 | [-0.56 - 0.67] | 0.85 |
| *20-25y* | 1.06 | [-0.21 - 2.33] | 0.09 | 0.53 | [-0.86 - 1.92] | 0.38 |
|  |  |  |  |  |  |  |
| *Association of patient reported anxiety and depression with HRV measures stratified by gender, cancer type, and age using linear regression models.  Models adjusted for age and cancer type when not the stratum of interest. Coefficients interpreted as change in PRO score for every 10msec change in HRV. CNS and non-CNS solid tumor categories collapsed for analysis. | | | | | | |
